# Developing Integrated Healthcare Models for Indigenous People: Insights from a Relational Systematic Scoping Review

**DOI:** 10.1007/s10900-025-01522-1

**Published:** 2025-10-16

**Authors:** Halina Clare, Edmund Wedam Kanmiki, Roxanne Bainbridge, Katrina Campbell, Clare Mangoyana, Stephanie Moriarty, Keighley-Tauariki Pascua, Carmel Nelson, Theresa Symes, Jenny Setchell

**Affiliations:** 1 Institute for Urban Indigenous Health (IUIH), 22 Cox Road, Windsor, QLD Australia; 2 Poche Centre for Indigenous Health, Faculty of Health, Medicine and Behavioural Science, The University of Queensland, Brisbane, QLD Australia; 3 ARC Centre of Excellence for Children and Families over the Life Course, The University of Queensland, Indooroopilly, QLD 4068 Australia; 4 ARC Centre of Excellence for Indigenous Futures, Faculty of Business, Economics and Law, The University of Queensland, Brisbane, QLD Australia; 5 Metro North Hospital and Health Service, Queensland Health, Brisbane, QLD Australia

**Keywords:** Indigenous health, Integrated healthcare, Service coordination, Health disparities, Holistic health

## Abstract

Integrated healthcare models show great promise for addressing health disparities affecting Indigenous people, which are often rooted in the enduring effects of colonisation. These models align with Indigenous holistic views of health, recognizing the importance of community, cultural knowledge, and connection to land. To understand how these models are being developed and implemented, we conducted a systematic scoping review. Guided by Indigenous methodologies and community needs, we searched four databases (Web of Science, PubMed, Scopus and ProQuest) for peer-reviewed literature on integrated healthcare for Indigenous communities in Australia, Canada, the United States, and New Zealand. Included articles were appraised using the Indigenous quality appraisal tool and analysed from a relational perspective supported by the Joanna Briggs Institute’s convergent integrated method. Nineteen publications met the inclusion criteria. Most studies were from Australia (53%) and Canada (26%), and most (74%) were published in the last five years, indicating a recent surge in interest. The review identified several key factors critical to the effective implementation of these models. These included strong community leadership and ownership, culturally and contextually relevant approaches, meaningful partnerships with stakeholders, and flexible service delivery. The review further highlights the importance of having motivated and well-trained health providers, as well as adequate funding. The wide variety of methods found in the studies reflects the complexity of integrated care and the influence of distinct cultural, disciplinary and contextual factors. The findings suggest that to improve healthcare and well-being for Indigenous populations, it is crucial to strategically address these key elements.

## Introduction

Despite the historical and ongoing impacts of colonisation, Indigenous people across the world continue to thrive, maintain cultural strengths and contribute actively to enhancing healthcare systems and practices [1]. Systemic inequities have undeniably contributed to persistent health disparities across Indigenous populations globally. In Australia, Canada, the United States of America and Aotearoa/New Zealand, for instance, the life expectancy of Indigenous people is between 5.5 and 10 years lower than that of non-Indigenous people [2–6]. This ongoing legacy of colonisation is unacceptable and there is an urgent need for change. Yet these realities do not define Indigenous people, whose knowledge systems and cultural practices continue to provide powerful, resilient frameworks for health and wellbeing [1, 7]. Across contexts, Indigenous conceptualisation of health extends beyond biomedical models [7, 8]. They encompass spiritual, emotional, physical, and relational wellbeing—grounded in connection to Country, community, and culture [1, 7, 8]. These holistic understandings offer invaluable contributions to contemporary health systems, especially when they are enacted through community-led integrated approaches.

While Indigenous-led approaches illuminate pathways to wellness, their full impact is often constrained by deeply rooted systemic barriers that continue to shape health outcomes. Addressing health disparities requires a systemic approach because the contributing factors to poorer health outcomes and wellbeing of Indigenous people include the ongoing colonial trauma, dispossession, forced removal from lands and harsh assimilation policies [3, 9]. Well-designed integrated healthcare models offer a promising approach to address some of these systemic concerns [10]. According to the World Health Organisation, integrated healthcare refers to an approach to healthcare provision that is coordinated and focuses on the whole person, including their physical, mental, and social needs [11]. It essentially involves a multidisciplinary team or teams and a person-centred approach with healthcare providers working together to create and implement personalised care plans for their clients [10, 11]. From an Indigenous standpoint, this approach to healthcare can open possibilities for care to be embedded in and driven by community and family priorities, needs and ways of knowing, being and doing [12]. Integrated care can support people and their communities to engage with a continuum of social supports, community engagement, health promotion, diagnosis, disease management, prevention, palliative and rehabilitation healthcare services. It thus aspires to provide a coordinated approach across various aspects of care services within and outside the health sector, and according to individual needs [11].

Integrated care approaches can align with Indigenous concepts of health and wellbeing which encompass a holistic view that considers the physical, mental, emotional, spiritual and social well-being of both the individual and the community and the interconnections with cultural practices, traditions and connection with land and nature [13]. It draws from the biopsychosocial-spiritual model that enjoins care providers to focus on a holistic approach to care by collecting and analysing information not only based on the biological aspects but also on the psychological, cultural, social and spiritual factors that underpin the health of individuals, families and communities as a whole [14]. The concept of integrated care has been applied in a variety of ways, ranging from structured care models to loosely defined coordinated approaches considering multiple vertical and horizontal systems affecting health and wellbeing [14].

Integrated healthcare can improve access to culturally responsive healthcare for Indigenous people and mitigate discrimination in service delivery [15]. These factors provide Indigenous people greater control and agency in how healthcare is designed and delivered. This is crucial for addressing the broader social determinants of health among Indigenous people [16]. The ultimate aim of integrated care models is to reduce health and access inequities, promote client agency and connect the wellbeing of individuals, families and communities [16, 17]. However, there is a paucity of knowledge on the critical success factors for developing and implementing effective integrated healthcare models with Indigenous people.

Arising from community-identified needs for effective integrated care services, this systematic scoping review examines the available peer-reviewed literature on healthcare integration, including the success factors for developing and implementing integrated service models for Indigenous communities. This paper contributes to addressing this community need for insights on this approach to healthcare and attends to this knowledge gap. The objectives of this paper are to: (1) examine the different types of integrated healthcare models used with Indigenous people, (2) synthesise the factors influencing the effective implementation of integrated healthcare with Indigenous communities and (3) identify and discuss critical success factors for the effective implementation of integrated healthcare with Indigenous communities.

## Methods and Materials

### Relational Search Methodology

A relational approach, grounded in Indigenous methodologies was used throughout this review process. Drawing from elements of Tynan and Bishop’s 2023 relational literature search approaches and other decolonising and Indigenous research methodologies [18, 19], we foregrounded relationships, respect and obligation in this research. The research questions that underpin this systematic review were raised by an Aboriginal and Torres Strait Islander Community-Controlled Health Organisation, the Institute for Urban Indigenous Health (IUIH). Community-Controlled Health Organisations in the context of Australia are Indigenous-led primary healthcare services governed by the communities they serve. The need came from the recognition that Aboriginal and Torres Strait Islander people who access healthcare through IUIH’s primary health services were not adequately informed nor receiving the care they needed to address health inequities arising from secondary, tertiary health and other non-community-controlled settings. This literature search began by building relationships amongst the key people, this included service providers and management within IUIH and non-community-controlled service providers, community members and university researchers. The author team reflects this diversity. Following this priority setting, there were multiple points of connection that helped steer the search direction, conduct, analysis and reporting – these are discussed below.

### Search Strategy

After relationally establishing the search parameters and terms informed by experiences of the community-controlled service provider, a systematic search was conducted on four electronic databases including Web of Science (core collection), PubMed, Scopus and ProQuest on 29th of June 2023 and an updated search conducted on 30th September 2024 for relevant articles reporting on models of integrated healthcare for Indigenous people. Broad terms were used to source as many relevant publications as possible. The full details of the search terms used are presented in Table 1. For example, terms such as Indigenous, American Indian, Metis, Inuit, Torres Strait Islander, Aboriginal, Māori, First Nations people were chosen to identify Indigenous people and terms such as integrated care, integration, primary healthcare, integrated hospital, community control care were used to identify the intervention/service type of interest. The search, screening and overall conduct of this review is guided by the Preferred Reporting Items for Systematic Reviews and Meta-Analyses (PRISMA) guidelines [20]. This review is registered with the international Prospective Register of Systematic Reviews (PROSPERO) with registration ID: CRD42024591046.

**Table 1:** Search terms used

Database Searched	PUBMED/Medline, Web of Science (core collection), SCOPUS, ProQuest,
Date of Search	29th June 2023, and updated on 30 September 2024
Population of Interest (AND)	Indigenous OR American OR indian OR native OR metis OR inut* OR torres strait islander OR maori OR aborigin* OR native hawaiian OR oceanic ancestry group OR australoid race OR pacific island america OR native OR north american amerind OR eskimo OR alaska native OR aleut OR inupiat OR kalaallit OR first nation OR kanaka OR maoli OR yupik
Interventions/Service Service type (AND)	delivery of health care OR integrated OR integrated care OR integration OR care model* OR service delivery OR health service* OR healthcare OR health care OR health-care OR integrated care OR care integration OR integration of care OR primary care OR primary health OR primary health care OR primary healthcare OR primary health-care OR health service OR community control service OR community care OR family care OR family medicine OR integrated hospital
Country	Australia OR New Zealand OR Canada OR United State of America OR USA OR America

### Study Inclusion and Exclusion Criteria

Inclusion and exclusion criteria were iteratively discussed amongst the multiple stakeholders, with additional connections and input sought as required. Studies were included if they were reporting on integrated models of care as defined by the World Health Organisation’s framework on integrated care as a holistic approach to healthcare provision including people-centred care, coordinated care services, and family/community-centred healthcare [21]. This broad definition was chosen to provide possibilities for family and community-led care as well as mainstream health system focussed care integration.

We included studies focused on integrated care for Indigenous people either for a whole system of care or for specific diseases. There were no constraints on publication date. Because English is the common language amongst the researchers, publications were excluded if they were not written in English. They were also excluded if they did not report on Indigenous people or health service integration. Inclusion was limited to studies from Australia, New Zealand, Canada and the United States of America (USA) because of similarities in the systemic exclusion of Indigenous people and underinvestment in holistic community-centred care in these countries.

### Screening and Data Extraction

**Fig. 1:**
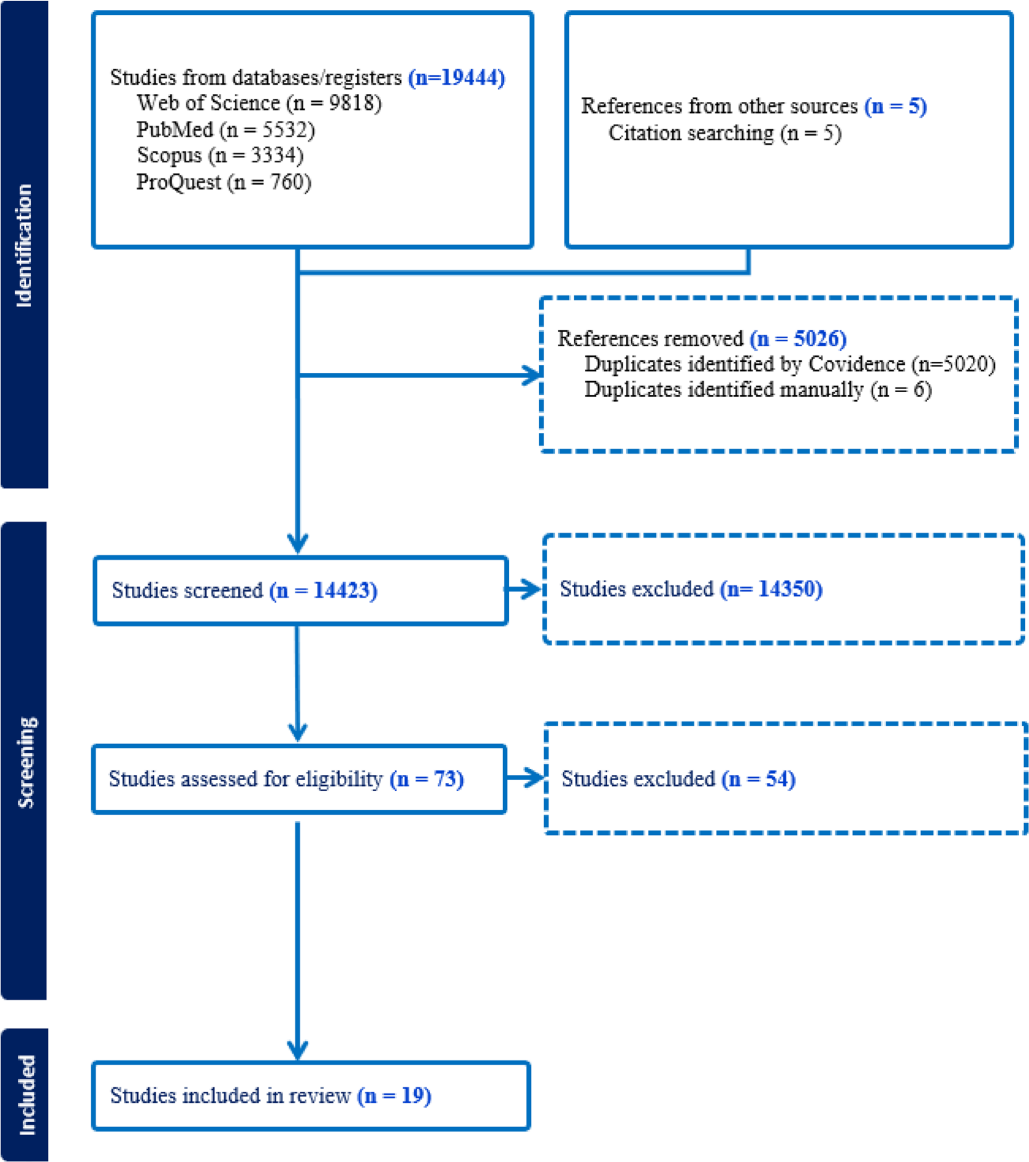
PRISMA flow chart of screening process

All publications retrieved from the database search were imported into *Covidence* systematic review software (Veritas Health Innovation, Melbourne, Australia) [22]. The initial screening involved removal of duplicates (done automatically by *Covidence software*). Initial screening was followed by title and abstract screening (by EWK and HC) and then a full article assessment based on the inclusion and exclusion criteria mentioned above (Fig. 1 presents the PRISMA flowchart showing the screening process). An iterative series of consultations with other team members and relevant community members ensured these processes aligned with the study aims and community needs (including all authors). The final publications included in the analysis are presented in Table 2. Information extracted from these publications included author names and publication year, country of study, study objective, methods, study participant characteristics, factors influencing integrated healthcare delivery and main findings/message.

**Table 2:** Characteristics of included studies

Author, Year	Country/People	Main Objective	Study Participants	Study Methods	Factors Influencing Integrated Care for Indigenous people	Main Findings
** Barnabe et al. 2017** [29]	Canada/First Nations, Metis, and Inuit people	Aim to evaluate an integrated model of care implemented to improve arthritis detection and treatment in an urban Indigenous population	A total of 38 patients were involved.	Quantitative study, pre- post-study design	Perception of discrimination during healthcare seeking based on education, income or race. Coordination of services by health professionals, support and encouragement by physicians, shared decision-making approach with physicians, Respect and equal treatment.	This healthcare model facilitated access for diagnosis and patients of inflammatory arthritis condition return to care. It also removed difficulties in accessing physician specialist while offering health care in an environment valued by Indigenous patients
** Carswell 2015** [35]	New Zealand/Māori	Outline a community-based program (Te Whiringa Ora) implemented to facilitate responsive, coordinated and seamless delivery of services for patients with chronic conditions and their families.	Enrolled 550 patients per annum	Case study, Implementation research	Many needs of patient, huge geographic spread, under resourced, patient follow-up required to ensure engagement of patients. Local providers unsure of program functions and thinking that it is taking their existing patients from them. Development of trust between GPs and program service providers/team Engagement and support of the hospital-based staff, strong cultural value base integration across agencies is key. Use of telehealth in monitoring facilitates early identification of clinical markers start and signs of acute episode for early intervention. Telehealth also facilitated communication between patient, GPs and secondary care specialists to coordinate the patients care. Availability of funding.	The program has considerable potential for improving healthcare delivery. In designing the program, wide consultations with primary care institutions, hospitals and GPs and community members were key.
** Chamberlain et al. 2016** [30]	Australia/Aboriginal people	Examine the utility of a care coordination framework for families with children 0–5 years.	Number of participants not provided	Qualitative study	Unclear accountability mechanisms, resource constraints, anxiety about transfer of information to child protection. frequent long waiting times cause difficulties, high turnover of staff in the sector could create challenges. Developing strong relationships and trust (interpersonal communication).	Relationships underpinned. care coordination.
** Curtis et al. 2024** [37]	New Zealand/Māori	To describe an Indigenous-led service delivery model implemented to improve integrated case and contact management of people with COVID-19.	About 46,000 Covid cases of which 97% were Māori	A case-study description	Poor understanding of Indigenous leadership by mainstream organisations, partnership and power-sharing by mainstream organisations. Shortages of staff with necessary attributes, recruitment delays and staff turnover. Training non-clinical Māori staff as contact tracers, partners in co-designing service provision, independent lines of accountability for Indigenous governance,	This model could be applicable to improving the design and delivery of public health services to other Indigenous and marginalized groups.
** Gorham et al. 2024** [38]	Australia/Aboriginal people	To describe a collaborative process for developing an integrated clinical decision support system to improve kidney patient care.	Able to link 11 ACCHS patient level data with 56 government primary health services and six hospitals.	Description of a collaborative partnership and co-design process	The absence of a unique identifier for linking patients’ data across multiple services. Some Patients have multiple aliases making it difficult to integrate data. Partnerships and effective governance pivotal.	Disconnected health services and separate EHRs result in information gaps and a health and safety risk, particularly for patients who access multiple health services.
** Henderson et al. 2023** [42]	Canada/First Nations, Metis, and Inuit people	Aimed to describe a program called Youth Wellness Hubs Ontario, integrated youth services program.	youth aged 12–25 years old (number not provided)	Narrative descriptive study	Strength-based approach improves innovation in program delivery and ensures services are provided in an interconnected way. Training staff to think holistically, program should target several aspects of care including mental health, substance abuse, school and/or work, relationships within the life of youth.	This program was found to be feasible in integrating mental health and early substance abuse interventions.
** Ivers et al. 2023** [44]	Australia/Aboriginal and Torres Strait Islander people	Identify barriers and facilitators to optimal integrated cancer care.	30 health professionals who deliver care to Aboriginal people	Qualitative study using semi-structured interviews	Unclear referral pathways, poor communication between patient and the treating team, and a lack of timely provision of discharge summaries. Availability of an integrated care team, presence of key health workers to help patients navigate the health system. Enhancement of the number of Aboriginal health professionals employed in all settings, funding for specialist appointments, and of electronic communication between primary care providers and specialist	Poor integration of primary and hospital care, in particular paper-based referral systems with no communication about appointments, and lack of communication back to primary care.
** Koski et al. 2017** [41]	Canada/First Nations, Metis, and Inuit people	Aim to describe and assess journey mapping procedure in Indigenous community of rural Canada through integration of indigenous health services and non-indigenous services.	8 community members involved in FGD, and Online survey	Participatory action research. Mixed method Qualitative & quantitative	Different views and expectations between healthcare providers and community the terminology used for the program must be crafted carefully. Building good relationships, effective communication and partnerships. Commitment of stakeholders is required to ensure success and continuity.	It was found that journey mapping enhances the integration of services and could be used be implemented in other Indigenous communities.
** Kowanko et al. 2009** [36]	Australia/Aboriginal and Torres Strait Islander people	Describe a program implemented to improve the coordination of care for Aboriginal people with mental disorders.	37 interviews with health and human service providers	Participatory action research Qualitative methods including document review, interviews and collaborative reviews.	Racism, confidentiality concerns, poor sharing of information, Lack of staff, service deficiencies, funding uncertainty, deficiencies of protocols, knowledge gaps among community and staff, Cultural understanding and acceptance, useful after hours/other services, Aboriginal health services and some general practitioners committed to holistic care, Efforts by local hospitals to provide culturally appropriate services, existence of clinical practice guidelines, Personalised transport help provided by Aboriginal health services.	The program showed that leadership within Aboriginal health services influenced healthcare coordination and overall sucess.
**Lee-Hammond 2013** [45]	Australia/Aboriginal and Torres Strait Islander people	Aim to report findings of research designed to consult members of the Noongar Aboriginal community in Perth, Western Australia, about integrated services for Aboriginal families and children	A total of 33 individuals participated in the study. 2/3 of participants were Aboriginal	Qualitative study	A culturally appropriate location is essential. The involvement of elders and other community members is required in the development of curriculum and service provision and governance Providers must understand community needs and respect Indigenous values, culture, traditions, and diverse child-rearing practices. Recognizing kinship networks and supporting holistic, community-based service delivery through appropriate planning, management, and review systems are also essential.	Paper concludes that the widely recognised need to ‘close the gap’ in Indigenous health and education services is not being met with sufficient funding and notes the ever-widening gap between purported policy imperatives and the process of addressing inequalities.
**Lewis et al. 2017** [15]	United States and Canada	Assess the effectiveness of integrated care models on Indigenous populations	N/A	Systematic review	Requires modification or changes to the delivery of care. Inculcating relevant cultural beliefs and practices improves positive outcomes. Sharing of knowledge and experiences between patients, community members and providers, continuous education for care teams and mentorship.	Integrated care models lead to a wide range of improvements including physical and mental health, reduction in substance abuse, improvements in employment, education and reduction in criminal justice involvement.
** Mann et al. 2021** [34]	Australia/Aboriginal and Torres Strait Islander people	This article details the effect of a community program that integrates care at primary-secondary interface on the rate of Emergency Department presentation and hospital admissions among older people with various health needs.	80 community members > 50yrs old	Quantitative, -Randomised control trial involving multicentre using a stepped wedge cluster study design	Requires careful planning and sufficient time to implement.	Evaluation of integrated multi sectorial healthcare programs need adequate preparation, and consideration of intervention period for effectiveness to be adequately measured.
** McCalman et al. 2017** [28]	Australia, Canada, New Zealand and United States/First Nations people	Examine the published research evidence on family-centred care interventions for First Nations early childhood health and wellbeing	Not applicable	Scoping review	Improve capacity of Indigenous workforce, promoting community cultural connectedness, support self-care and improves links with clinic. Compassionate service delivery, flexibility of accessing care and continuity.	Early childhood and parental/care givers health outcomes of Indigenous people are improved by family centred interventions. Family-centred care also improves satisfaction and access to mainstream services.
** McCalman et al. 2023** [31]	Australia/Aboriginal and Torres Strait Islander people	Examines the efforts of an Aboriginal and Torres Strait Islander community—Yarrabah in north Queensland to develop integration strategies for mental health and wellbeing service improvements for school-aged youth(5–18years).	32 participants, all youth aged 11–24 years old and 24 service participants	Community-based participatory research	Disjunct between youth need and service provision, youth reluctance to seek help due to community expectation of youth resilience and strength. Access to youth facilities, spaces and activities, safe and available points of contact, listening to youth, linking with community members, providing wellbeing promotion programs, safety, trust, relationality and consistency, cultural safety.	Need for ongoing dialogue as the basis for co-designing and implementing improvements to wellbeing supports and mental health services for Indigenous youth.
** Osborn et al. 2022** [32]	Australia/Aboriginal and Torres Strait Islander people	Examine the availability, accessibility and utilisation of comprehensive community-based health services for Indigenous people. And explain the factors contributing to effective provision of health services.	11 participants, across 3 local health services (*n* = 4), 2 of whom were from an ACCHO, 2 local schools (*n* = 5), and 2 FIFO services (*n* = 2)	Case study, mixed-methods methodology	Lack of funding and resources problems, lack of coordination on service availability and locations. Inadequate incentives for cooperation and resources sharing. Lack of collaboration among agencies Relationships built on trust is key especially for vulnerable people who are likely to fear due to ill-health challenges. Training and capacity building for staff health workers.	Providing local services with resources, training, and capacity building improves effectiveness of service delivery and continuity of patient care.
** Rooney et al. 2023** [39]	Australia/Aboriginal and Torres Strait Islander people	Conduct an integrative literature review to reveal any evidence supportive of the integration of traditional therapies for First Nations people in Australia within a Western healthcare model.	Not Applicable	Integrative literature review	Fear of being judged or stigmatised, lack of understanding of the bioactive components of traditional medicines and the interactions they may have with Western medicines, culturally safe and appropriate care, inclusion and recognition of traditional healers as legitimate health practitioners, a two-way healthcare system between Western medical practitioners and First Nations traditional practitioners. building collaborative communication between Western healthcare providers and First Nations people, thus enhancing trust and rapport between clinicians and First Nations clients.	There is need to include traditional therapies within a Western healthcare system. Creating a culturally safer and appropriate healthcare experience for First Nations people in Australia will contribute to advancement in decolonising current healthcare models
** Sasakamoose et al. 2024** [40]	Canada/First Nations, Metis, and Inuit people	To examine the integration of Justice, Diversity, Equity, and Inclusion principles and cultural responsiveness in fostering community resilience and mental wellbeing.	Not Applicable	Mixed-methods approach, case study	Not acknowledging the land as a source of healing, wisdom, and identity Commitment to spiritual and cultural practices as vital healing components, culturally respectful, community-driven health interventions, importance of land and cultural practices in healing, spaces that respect traditional values.	Holistic, healing families rather than individuals, integrating various health, mental health, and wellness services.
** Simmons 2003** [33]	Australia/Aboriginal and Torres Strait Islander people	To describe the effectiveness of an integrated primary–secondary care diabetes clinic on metabolic control among Indigenous patients in a rural community	47 patients (39 with type 2 diabetes, one with type 1 diabetes and seven with gestational diabetes	Retrospective cohort study	Significant resources required, financial barriers to implementing modern diabetes care include the out-of-pocket expenses for orlistat and nicotine patches.	The introduction of an integrated diabetes scare service in an Indigenous health service can overcome many of the pre-existing barriers to achieving metabolic targets
** Wu et al. 2023** [43]	Canada/First Nations, Metis, and Inuit people	Aim to describe lessons, disconnectedness, and experiences of current integrated care programs. for Indigenous adults in Canada	Interviews with 9 programs serving Indigenous people	Qualitative	Tensions and disconnections with culture Community involvement, engagement and commitment are key. Focusing on addressing communities’ needs and goals, incorporating culture in to program development and delivery, good leadership is integral, and attention to collectivism community cultural healing instead of individualism. Continuous evaluation.	Partnerships that are Indigenous led are important in promoting and implementing integrated care because such partnerships leverage indigenous ways and knowledge to achieve equity in health in an integrated care system.

### Data Analysis and Synthesis

#### Quality Appraisal

The Indigenous Quality Appraisal Tool (QAT) developed by Aboriginal and Torres Strait Islander researchers (Harfield and colleagues) was employed in appraising the quality of included studies [23]. This tool was designed using Nominal Group and Delphi techniques for evaluating the quality of research and programs from an Indigenous perspective, emphasising the importance of community engagement; cultural safety with key considerations including the extent to which studies incorporate Indigenous ways of knowing; and whether the research design reflects Indigenous community priorities. This 14-question appraisal tool was selected by our research team and Indigenous service providers as the most appropriate tool for evaluating the quality of research publications involved in this review because it enabled us to understand whether the research was thoroughly addressing Indigenous people’s needs and priorities. CM and EWK independently scored and then discussed to reach an agreement on all included studies – with high level input from HC, JS, SM and KP. Studies scored a maximum of two points if they adequately satisfied a criterion; one if partially addressed, and zero if there was no indication that it was addressed. Points were then summed and ranked. Studies were classified into three categories based on their relative score, 0.0–0.4 for low quality, 0.5–0.7 for medium quality and 0.8–1.0 for high quality. This approach has been used by other systematic reviews [24, 25] and can be a helpful way of assessing relative quality among studies involved in systematic review and give a sense of the overall quality of the available literature. Our scoring aimed to provide a relative ranking of studies within the context of this review. Like any quality criteria, it did not aim to address all quality concerns, particularly across a range of methodologies.

#### Evidence Synthesis

Included articles were analysed and synthesised from a relational perspective supported by the convergent integrated method developed by the Joanna Briggs Institute for systematic reviews [26]. The convergent integrated approach combines quantitative and qualitative data to provide a comprehensive understanding of a phenomenon. In this approach, the quantitative and qualitative data were brought together and analysed and integrated into a single set of findings to provide insights into the review topic [26, 27]. As a first step, data was extracted by EWK from all study types (quantitative, qualitative, mix methods and review articles) into a “qualitised” fashion through a narrative interpretation of findings in text. A draft of this narrative interpretation was discussed in a group interpretation meeting involving Indigenous community members, Indigenous health service providers from the partnership, university and community-controlled researchers. The meeting provided an opportunity to relationally contribute to the interpretation and to produce findings that are culturally, locally and broadly meaningful in relation to Indigenous people. All authors contributed to the finalisation of the interpretation and reporting of results.

## Results

### Search Outcome and Screening

Figure 1, the PRISMA flow diagram, outlines the search and screening process. The initial broad-scope search across four databases and supplementary hand searches generated 19,449 studies. First, 5,026 records were identified as duplicates and removed by *Covidence software*, leaving 14,423 publications for title and abstract screening. This resulted in 14,350 being removed. The remaining 73 publications went through full article screening based on the inclusion and exclusion criteria mentioned above. Nineteen publications met the inclusion criteria and were included in this review.

### Study Characteristics

Table 1 presents information on the background characteristics of the 19 studies included in this review after screening. All studies were published between 2003 and 2024, although almost all (74%, 14) were published within the last five years (2017–2024). Most studies were conducted in Australia (53%, 10), followed by Canada (26%, 5). Two studies were conducted in New Zealand and another two were multi-country including Australia, Canada, the USA and New Zealand [15, 28].

### Study Objectives of the Included Studies

Publications included in this review had a variety of study objectives and contexts. For instance, seven publications aimed to develop and/or evaluate existing integrated models of care and identify factors contributing to effective service provision [15, 29–34]. Four of the included publications aimed to describe the implementation of an integrated care program [35–38], while three examined existing evidence on related integrated service models like culturally tailored family-centred care with Indigenous populations [28, 39, 40]. One study however aimed to provide both a description of the intervention and an evaluation of its outcomes [41]. Furthermore, two publications aimed to describe lessons learnt, strengths, opportunities and knowledge from implementing integrated models of care with Indigenous populations [42, 43], and one explored barriers and facilitators to accessing integrated healthcare [44]. There was also one study focused on engaging communities to understand the types of services they wanted and their preferences for modes of service delivery [45].

### Study Designs and Methods

There were a variety of qualitative, quantitative, mixed methods and reviews included. Eight publications were qualitative studies involving either interviews, focus group types of discussions (yarning circles), observations or document reviews [30, 31, 36–38, 43–45]. Three studies were purely quantitative including either pre-post evaluations, randomised control trials and cohort study designs [29, 33, 34]. Four studies were mixed methods mostly using implementation research approaches and/or case studies [32, 35, 40, 41]. There were also four reviews, each with different methods, namely: systematic review, scoping review, narrative synthesis and a literature review [15, 28, 39, 42].

### Target sub-populations

All included studies involved integrated healthcare programs for Indigenous people given that this was a key inclusion criterion. However, there were specific sub-populations of Indigenous people in many of the studies such as: children, youth and older adults. Two publications reported on interventions or programs for youth [31, 42], another two focused on families or women with children under 5 years [28, 30], one focused on adults [40], and another on older Indigenous people [34]. Others focused on Indigenous people in particular geographic settings such as urban or rural locations.

In terms of health foci, five publications focussed on Indigenous people (and their families) with chronic conditions including such as arthritis, diabetes, cancer and kidney disease [29, 33, 35, 38, 44], two considered Indigenous people with substance use or psychosocial wellbeing concerns [31, 36] and one focused on case and contact management for COVID-19 cases in Indigenous people [37]. Five studies did not indicate a specific sub-population of interest [15, 32, 39, 40, 45].

### Quality of Studies Based on Indigenous Quality Appraisal

Table 3 provides the combined quality appraisal scores of included studies. None of the studies scored all points out of a possible 28, with the scores range from 6 to 25 (median = 17 points). Eight (42%) of the 19 studies scored 0.8 or higher and were regarded as high-quality studies [28, 29, 31, 32, 36, 37, 40, 43], a further eight studies were classified as medium quality [15, 30, 33, 38, 41, 42, 44, 45] and three (16%) studies were considered low-quality [34, 35, 39].

**Table 3:** Quality assessment scores

Study	Respond to needs/priority determined by Indigenous community	Community consultation and engagement appropriately inclusive	Indigenous research leadership	Indigenous governance	Local community protocols respected and followed	Agreements in regard to rights of access to Indigenous existing intellectual and cultural property	Agreements to protect Indigenous people ownership of intellectual and cultural property	Indgenous peoples control over collection/management of research materials	guided by an Indigenous research paradigm	Strengths-based approach,	Plans to translate the findings into sustainable changes in policy and/or practice	Benefit the participants and Indigenous community communities	Demonstrate capacity strengthening for Indigenous individuals	Did everyone involved in the research have opportunities to learn from each other?	Total Score	Relative score
** Barnabe et al. 2017**	2	1	2	2	1	1	2	1	1	1	0	2	2	2	20	0.8
** Carswell 2015**	1.5	0	0	0	0.5	0	0	0	0.5	0.5	2	1	1	0	7	0.3
** Chamberlain et al. 2016**	1	1	1	1	0.5	0	0	1.5	1.5	2	1	2	0.5	0.5	13.5	0.5
** Curtis et al. 2024**	2	1.5	2	2	2	0.5	0.5	2	2	2	2	2	0.5	0.5	21.5	0.8
** Gorham et al. 2024**	1.5	2	0.5	2	0	2	2	1	0	0	2	2	0.5	1	16.5	0.6
** Henderson et al. 2023**	2	2	0	0	0.5	0	0	0.5	1.5	1.5	1.5	2	1	1	13.5	0.5
** Ivers et al. 2023**	2	2	2	2	1.5	0	0	2	1	1	1	1	1.5	1	18	0.7
** Koski et al. 2017**	2	1	2	1	1	0	0	0.5	1	1.2	1.5	1.5	1	1	14.7	0.6
** Kowanko et al. 2009**	2	2	2	2	1	0	0	2	2	1	2	2	2	1	21	0.8
**Lee-Hammond 2013**	1	2	0	1	2	0	0	1.5	2	2	1.5	2	1	1	17	0.7
**Lewis et al. 2017**	1	0	2	2	0	0	0	1	0	1.5	1.5	1.5	1	1	12.5	0.5
** Mann et al. 2021**	1	1	0	0.5	1	0.5	0.5	0	0.5	0	0	0.5	0	0.5	6	0.2
** McCalman et al. 2017**	2	1	2	2	1.5	1.5	1	1.5	2	1.5	2	2	1.5	0.5	22	0.9
** McCalman et al. 2023**	2	2	2	1.5	2	1	1	1	1.5	1.5	1	2	1.5	1.5	21.5	0.8
** Osborn et al. 2022**	2	1.5	2	2	2	0.5	0.5	1.5	0.5	2	1.5	2	2	0.5	20.5	0.8
** Rooney et al. 2023**	1	0	0	0	0	0	0	0	1	1	0.5	1.5	1	1	7	0.3
** Sasakamoose et al. 2024**	2	2	2	2	1	2	2	2	2	2	1.5	2	1.5	1.5	25.5	1.0
** Simmons 2003**	1.5	1.5	0	0	1.5	0	0	0.5	5	1	1	2	0.5	0.5	15	0.6
** Wu et al. 2023**	1.5	1.5	2	2	1.5	0	0	2	2	2	2	2	1	1	20.5	0.8

Almost all included studies were responding to identified needs or priorities of Indigenous communities or organisations and demonstrated appreciable community engagement except for three studies where there was no indication of any Indigenous community engagement [15, 35, 39]. Six studies scored very low on Indigenous research leadership and governance due to their lack of involvement of Indigenous authors or inadequate demonstration of Indigenous oversight over their research [33–35, 39, 42, 45]. Eleven studies did not provide any indication of having an agreement regarding Indigenous peoples’ rights and ownership of existing intellectual and cultural property [15, 30, 33, 35, 36, 39, 41–45]. Further, five studies did not provide clear indication of being guided by Indigenous research methods and paradigms [15, 32, 34, 35, 38]. Three studies had virtually no indication of plans to translate findings [29, 34, 39], but all included studies had contributions that could lead to benefits for Indigenous people. Overall, this appraisal suggests that the research evaluated is of medium quality when appraised from an Indigenous perspective – there is consistent engagement with some of the key criteria but there is considerable room for improvement in future studies. Drawing from the findings of the quality appraisal, to enhance the trustworthiness of the thematic results reported below, we reduced the focus on the studies with low quality ratings.

### Factors Influencing the Successful Development and Implementation of Integrated Healthcare Models for Indigenous People

**Fig. 2:**
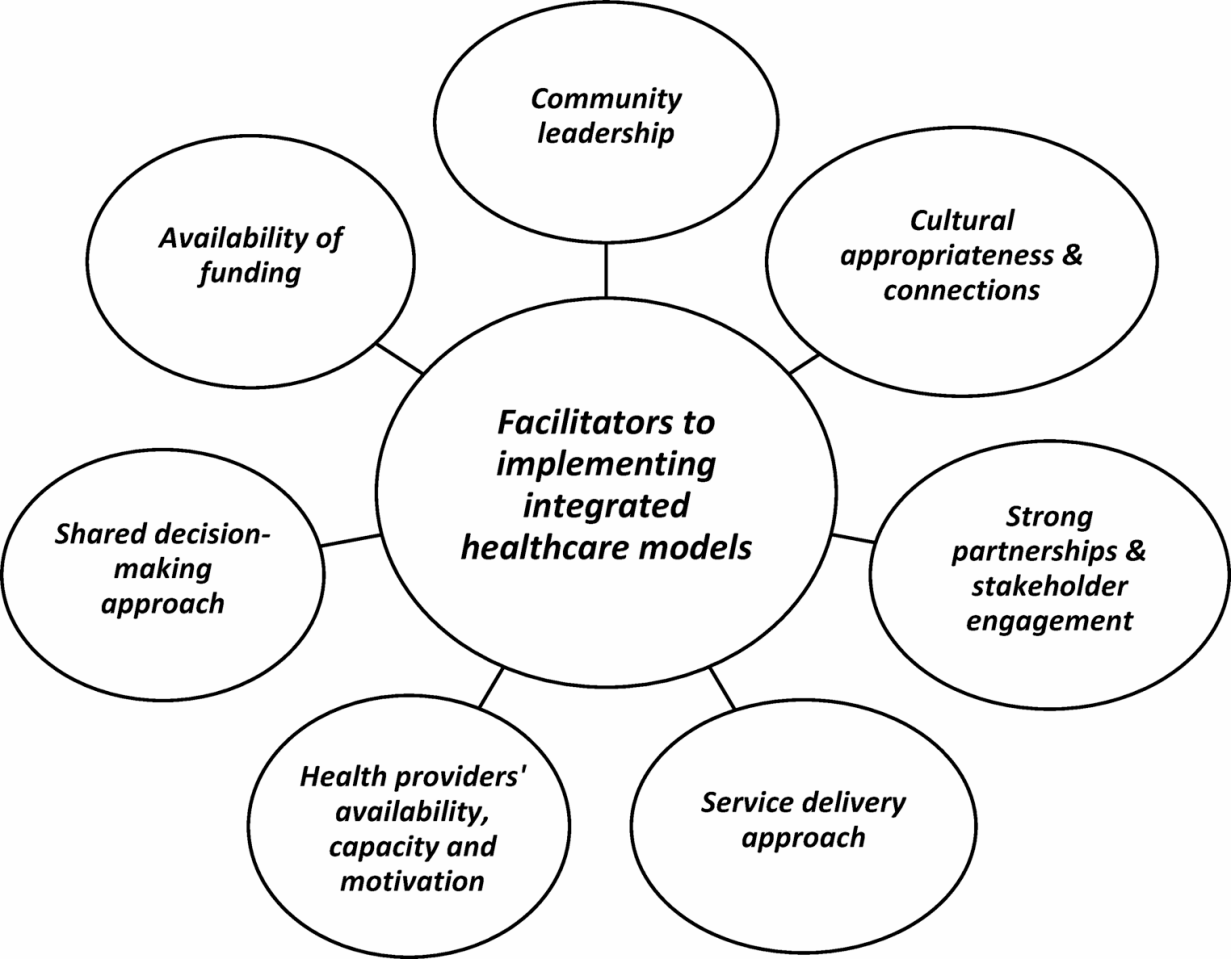
Facilitators to effective implementation of integrated healthcare models with Indigenous people

**Fig. 3:**
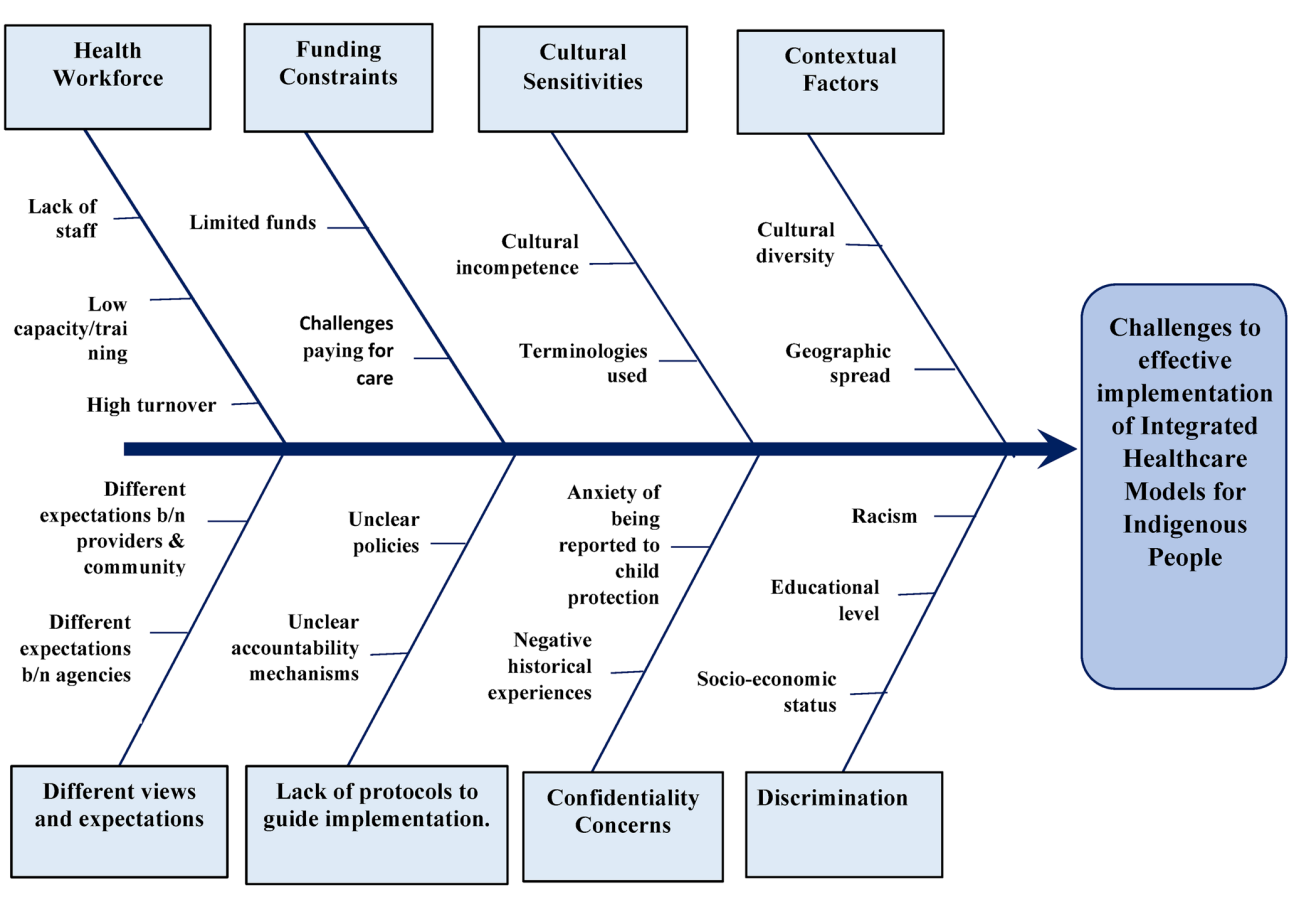
Challenges to effective development and implementation of integrated healthcare models

Figures 2 and 3 show the facilitators and challenges respectively, that were identified from the included studies for the effective development and implementation of integrated healthcare models for Indigenous people. The following broad themes were used to jointly present the factors identified to influence integrated healthcare models for Indigenous people: community leadership and ownership; cultural and contextual factors; partnerships and stakeholder engagement; service delivery approach; health providers’ availability, capacity and motivation; and adequate funding.

#### Community Leadership and Ownership

A strong facilitator of effective health integration programs was committed and active Indigenous leaders who were interested and involved in the integrated care services. For example, a program with Aboriginal people with mental health and/or substance use challenges living in the Eyre Peninsula region of South Australia showed that leadership within Aboriginal health services influenced progress [36]. Further, community needs and goals should determine the type and range of services to provide [43]. For example, a study with First Nations, Metis and Inuit people in Canada about adult mental health services suggested that community needs can be achieved by ensuring community leadership determines the services that are most appropriate for their communities [43]. Māori community ownership of programs was found to lead to very positive outcomes in integrated care in the urban setting of Eastern Bay of Plenty in New Zealand [29].

**Cultural and contextual factors**: Foregrounding Indigenous cultural values, traditions, spiritual connections and family and community in health provision and integration processes facilitated successful program implementation [39, 40]. For instance, a program aimed at improving care for Aboriginal people in Eyre Peninsula of South Australia who have mental health disorders and challenges related to substance use found that strong cultural knowledge, connections to community amongst program staff and service providers facilitates culturally appropriate integrated services [36]. Similarly, a systematic review of studies among Indigenous communities from the USA and Canada highlighted how the inculcation of cultural beliefs and practices in healthcare workplaces improved cultural connectedness and cultural safety ultimately resulting in positive outcomes for Indigenous people and their communities engaging with integrated care services [15]. Language use was also important: a study aiming to improve the quality of palliative home care through service integration in a rural Indigenous community in Canada highlighted the need for appropriate terminologies that are culturally acceptable in each context [41]. How programs were framed and presented to communities was also key, as a program targeting youth found reluctance of young people to participate because of communities’ cultural expectations that youth ought to be resilient and strong [31]. These and other cultural insensitivities are legacies of colonisation that continue to impact integrated and effective healthcare provision and require systemic change to address.

#### Partnerships and Stakeholder Engagement

Commitment of stakeholders is required to ensure the success and continuity of programs [41]. Strong partnership and stakeholder engagement to building trust and relationships at individual and organisational levels was highlighted by several studies [30–32, 41]. Good partnership and engagement can prevent misinterpretation and unclear expectations which can challenge the implementation of integrated care when healthcare providers and community members have different expectations of the program [41]. For example, a study in a rural community within the Eastern Bay of Plenty in New Zealand, found that some service providers had the incorrect perception that an integrated program would divert Māori patients from existing services [35]. These beliefs can inhibit their cooperation and support. The same study also demonstrated that this issue can be improved by building trust and mutual understanding of program objectives [35]. Aligning or including varied needs, expectations and perspectives can help address these types of barriers. Indigenous community-led partnerships are important in promoting and implementing integrated care because they leverage Indigenous ways of being, knowing and doing to achieve health and wellbeing in an integrated care system [40, 43].

#### Values Underpinning Service Delivery

The values driving care delivery are important to the success of programs. For Indigenous people, adopting a strength-based approach to care delivery is important because it counters negative stereotypes and health assumptions. This approach is clarified in a study developing and implementing integrated Indigenous youth services in Ontario, Canada [42]. The need for a holistic and collective approach to care and well-being for the individual, family and community was highlighted [36, 45]. Also, principles of flexibility were important. For example, allowing for home care options, after-hours services for community members, continuity and family-centred and flexibility for including traditional healing/spiritual help was found to improve service integration in a scoping review of studies conducted in Australia, Canada, USA and New Zealand [28]. Being ethical and trustworthy were also key values, such as ensuring timely and appropriate delivery of care where privacy and confidentiality are assured [36]. For instance, an Australian study found that Aboriginal community members had confidentiality concerns and anxiety that program workers would share their private information with child protection or other state actors which can led to forced separation of families [30]. Values driven care is of particular importance to people who have experienced enduring marginalisation.

#### Structural Support and Processes

It is important that service delivery values and approaches are embedded in program/clinical practice guidelines, policies, and procedures to effectively guide program staff and stakeholders [36]. Systemic discrimination based on education level, socioeconomic status, or race significantly hinders the successful implementation of service integration models [29, 36, 46]. For instance, an Australian study highlighted racism as a prevalent discriminatory factor [36]. This review also found that implementing integrated healthcare models for Aboriginal and Torres Strait Islander people in Australia is particularly challenging because of a lack of guiding protocols for service integration and knowledge gaps among both community members and healthcare staff. The absence of clear protocols can contribute to the unclear accountability mechanisms identified in another study [30], as well as poor coordination and collaboration between agencies and stakeholders, potentially exacerbated by inadequate incentives for cooperation [32].

#### Workforce Capacity and Capability

Healthcare providers and other staff are key to the successful implementation of service integration. Recruiting staff from within Indigenous communities enhances the cultural leadership, knowledge and practices of the workplace and improves community connection and service use [44]. Also important is improving the capacity of staff through training that fosters inter-professional collaboration and a holistic service approach [28, 32, 42]. Staff must understand and respect the needs and aspirations of communities they serve [45], and adopt a strong equity-focus [29]. Other specific issues raised in the literature around workforce included their levels of experience, how they are oriented into the workplace, a lack of staff and high staff turnover [30, 36]. Without sufficient lived experience or training, language used by staff is sometimes not culturally appropriate and can produce stigma and shame for community members, hampering good integrated care [41]. The people involved in integrated care are core to its work and must have the relevant social, cultural and technical skills for successful service delivery.

#### Adequate Resourcing

Sufficient funding and resources are essential for effective integrated healthcare delivery, supporting service provision, personnel costs, infrastructure development and medical supplies [35, 45]. A study on the delivery of community-based integrated coordinated service amongst Māori clients with chronic conditions in New Zealand noted that effective and continuous engagement of community members often required different resources than those funded within mainstream settings [35]. Research on integrated cancer care within Aboriginal and Torres Strait Islander communities in Australia highlighted the need for funding to support specialist staff and facilitate electronic communication mechanisms between primary and secondary service providers [44]. Resourcing care for accessibility is also key – for example, ensuring or providing transportation to enable access to care within an integrated system [36]. Access to services can also be limited due to the significant service costs for clients, as was observed in a study of integrated care for Aboriginal and Torres Strait Islander diabetes clients in Australia [33]. Under-resourcing resulted in service deficiencies despite providers striving to address the varied health needs of the population within an integrated healthcare program [35, 36]. Sufficient financial and other resources are pivotal in any effort to develop and implement integrated healthcare programs.

Figure 3 presents a fishbone diagram showing the key challenges this review identified to the effective development and implementation of integrated healthcare models for Indigenous people synthesised from the reviewed publications. 

## Discussion

Led by a community-controlled organisation’s need for more information about integrated care models, this systematic scoping review examined factors influencing the effective development and implementation of integrated models of care for Indigenous people worldwide. Integrated healthcare models have demonstrated a wide range of improvements for Indigenous people including improved mental and physical health, reduced alcohol and substance abuse, reduced criminalisation and better educational outcomes [15]. This review analysed the existing evidence to identify key factors for the successful development and implementation of integrated healthcare for Indigenous people and considered possible challenges.

Nineteen publications were included in this review, with 74% of these published within the last five years, indicating a growing body of research and interest in integrated healthcare models for Indigenous people. This finding highlights an increasing understanding of the persistent health disparities faced by Indigenous people and the potential of integrated healthcare to contribute to address these inequities. Included publications used a wide variety of study methods and were implemented in a variety of settings. They also targeted varied Indigenous sub-populations like children, youth, older adults or disease-specific targets although some also targeted the general population of Indigenous people. The diversity of implementation strategies across included studies reflects the complexity of integrated healthcare and the need for multifaceted and adaptable approaches tailored at specific contexts.

Several interconnected factors were identified as influencing the effective development and implementation of integrated healthcare models for Indigenous people. Community leadership and ownership are among these crucial factors [47–49]. This highlights the importance of self-determination of Indigenous people in healthcare planning and delivery [50]. Thus, empowering communities to lead and own the process facilitates culturally appropriate services that address the local needs of communities and ensure sustainability of programs [49, 51, 52]. Closely related to this is the importance of cultural and contextual factors in developing and implementing integrated health care for Indigenous people [53]. We found that allowing flexibility for integrating traditional healing practices, respecting Indigenous knowledge systems and adapting services to the specific cultural context of each community is essential for building trust and ensuring service utilisation within an integrated healthcare model [41, 43, 49, 52, 54]. Ignoring these factors can lead to ineffective interventions and perpetuate existing health disparities.

Partnerships and stakeholder engagement are also pivotal to effective development and implementation of integrated healthcare [38, 53]. Building strong relationships between healthcare providers, community members and organisations, government agencies and other relevant stakeholders promotes collaboration, resource sharing and a common understanding of community health needs [53]. Effective engagement ensures integrated care programs are designed and implemented in a collaborative and coordinated manner so that diverse perspectives are factored into the process [35].

The service delivery approach is very important. Models that prioritise holistic care, address social determinants of health, and integrate primary care with specialised services are more likely to achieve positive outcomes. Services ought to be accessible to all Indigenous people within the targeted communities, regardless of where they live, their socio-economic situation or their educational background [17, 55, 56]. Measures should be put in place to avoid all forms of discrimination and racism [29, 36, 46]. Service coordination is key to service integration. An integrated healthcare program should involve a core element of care coordination across different services and providers. Care coordination enhances patient outcomes, improves the quality and continuity of care, increases healthcare system efficiency, supports effective management of chronic conditions, promotes health equity, strengthens communication among providers and improves patient and caregiver satisfaction through an integrated and patient-centred approach.

Health providers’ availability, capacity and motivation are key factors to consider in the implementation of integrated care. Recruiting and retaining qualified providers, particularly those from Indigenous communities, is essential [52]. Training and professional development activities are required to enhance health workers’ capacity in areas such as cultural competency, interprofessional collaboration, and holistic care [52, 57]. A motivated workforce, committed to serving Indigenous populations, is crucial for successful implementation. Finally, adequate funding is a prerequisite for all other factors. Sustainable funding is necessary to support staffing, infrastructure, program development and ongoing evaluation. Without sufficient resources, even the most well-designed integrated care models will struggle to achieve their intended impact.

To ensure an integrated healthcare program is successful, it should begin by establishing a community steering committee or group involving local community leaders, respected community members, representatives from health and social service organisations and other key stakeholders [57]. The steering committee should be responsible for overseeing the development and implementation of the program, and a needs assessment should be conducted at its inception to identify the health and social well-being needs of the targeted Indigenous community. This information will facilitate the development of a program that meets the needs of the community.

When developing healthcare integration program, plans and protocols, they should outline the goals, objectives and strategies of the program. They should include an explicit framework of key program activities, assign responsibilities, set timelines and a budget to avoid ambiguity during implementation. The program should be phased in slowly, allowing for stakeholder participation while building community trust gradually. A phased approach helps to ensure that the program is implemented smoothly, and any challenges can be addressed early on [58]. Lastly and importantly, is the need for routine monitoring and evaluation of the program to help ensure that the program is meeting its goals and objectives and that any necessary adjustments can be made [34, 43]. Flexibility and adaptability are key to program success.

### Strengths and Limitations

This review’s strength is its focused on integrated healthcare for Indigenous peoples across four countries: Australia, Canada, the USA, and New Zealand. While their healthcare systems vary, they share a common history of systemic exclusion and ongoing health disparities for Indigenous populations. This specific focus allows for a deeper, more relevant analysis of how integrated care models function within similar contexts.

Key methodological strengths include the use of a relational perspective and the Indigenous quality appraisal tool (QAT). This tool provided a unique and culturally grounded perspective, valuing Indigenous leadership and research methods. It ensures the evaluation of research quality is culturally relevant and not based solely on mainstream academic standards.

A limitation in this review is the intentional exclusion of other nations, which means some relevant studies may have been missed. Also, while the QAT is a strength, it is not a comprehensive measure of all research quality, as it prioritizes certain cultural elements. The findings also suggest that despite good overall scores, there is room for improvement in future research, particularly in ensuring Indigenous leadership and culturally appropriate methods are at the forefront.

## Conclusion

This systematic scoping review assesses the available evidence on developing and implementing integrated healthcare programs for Indigenous people. Findings demonstrate a growing body of research on integrated healthcare models for Indigenous people. However, the diverse approaches employed across studies also highlight the inherent complexity of integrated healthcare. There is no one-size-fits-all solution, and the development and implementation of effective integrated healthcare requires multifaceted approaches tailored to the specific needs and contexts of individual Indigenous communities.

Our findings show that successful integration hinges on an interplay of several interconnected factors, including community leadership and ownership, cultural appropriateness, partnerships, service delivery approach, skilled and motivated workforce, and adequate funding. These factors are not isolated but rather mutually reinforcing components of a successful integrated care system. Thus, a holistic and systems-based approach is required in developing and implementing successful integrated healthcare models that can effectively contribute to improving health equity for Indigenous populations.

## Data Availability

All data generated or analysed during this study are included in this published article and its supplementary information files.
